# The Nordic Maintenance Care Program - Time intervals between treatments of patients with low back pain: how close and who decides?

**DOI:** 10.1186/1746-1340-18-5

**Published:** 2010-03-08

**Authors:** Kjerstin F Sandnes, Charlotte Bjørnstad, Charlotte Leboeuf-Yde, Lise Hestbaek

**Affiliations:** 1 Institute of Clinical Biomechanics, University of Southern Denmark, Odense, Denmark; 2 Nordic Institute for Chiropractic and Clinical Biomechanics, University of Southern Denmark, Odense, Denmark

## Abstract

**Background:**

The management of chiropractic patients with acute and chronic/persistent conditions probably differs. However, little is known on this subject. There is, for example, a dearth of information on maintenance care (MC). Thus it is not known if patients on MC are coerced to partake in a program of frequent treatments over a long period of time, or if they are actively involved in designing their own individualized treatment program.

**Objectives:**

It was the purpose of this study to investigate how chiropractic patients with low back pain were scheduled for treatment, with special emphasis on MC. The specific research questions were: 1. How many patients are on maintenance care? 2) Are there specific patterns of intervals between treatments for patients and, if so, do they differ between MC patients and non-MC patients? 3. Who decides on the next treatment, the patient, the chiropractor or both, and are there any differences between MC patients and non-MC patients?

**Methods:**

Chiropractic students, who during their summer holidays were observers in chiropractic clinics in Norway and Denmark, recorded whether patients were classified by the treating chiropractor as a MC-patient or not, dates for last and subsequent visits, and made a judgement on whether the patient or the chiropractor decided on the next appointment.

**Results:**

Observers in the study were 16 out of 30 available students. They collected data on 868 patients from 15 Danish and 13 Norwegian chiropractors. Twenty-two percent and 26%, respectively, were classified as MC patients. Non-MC patients were most frequently seen within 1 week. For MC patients, the previous visit was most often 2-4 weeks prior to the actual visit, and the next appointment between 1 and 3 months. This indicates a gradual increase in intervals. The decision of the next visit was mainly made by the chiropractor, also for MC patients. However, the study samples of chiropractors appear not to be representative of the general Danish and Norwegian chiropractic profession and the patients may also have been non-representative.

**Conclusion:**

There were two distinctly different patterns for the time period between visits for MC patients and non-MC patients. For non-MC patients, the most frequent interval between visits was one week and for MC patients, the period was typically between two weeks and three months. It was primarily the chiropractor who made the next visit-decision. However, these results can perhaps not be extrapolated to other groups of patients and chiropractors.

## Background

A considerable proportion of patients seeking chiropractic care for low back pain suffer from relatively long-lasting problems [1,2]. Some of these are treated only in their acute phase, whereas others receive more prolonged care. This could be to prevent new episodes of pain that are likely to occur, because of the recurring nature of low back pain. Among chiropractors, secondary and tertiary prevention is called maintenance care (MC). Although MC appears to be relatively commonly used among chiropractors, the prevalence with which maintenance care is used has not been established, not much is known about it [3], and its efficacy has been tested only in a pilot study [4].

Attempts have been made to obtain information on chiropractors' use of MC for patients with low back pain. It has, for example, been established that there seems to be relative consensus on its indications and non-indications but it is not known how frequently such patients are seen. In a study by Jamison, Australian chiropractors were asked with which time interval they saw such patients. The responses went from once a month to once every three to four months [5]. However, the response rate in this study was only 22% and the results were based on subjective reports. To our knowledge, no other serious attempt has been made to establish the visit patterns of MC patients.

To study this closer, it would be relevant to establish if treatment scheduling of MC patients is indeed distinctly different from that of other patients. This should be done objectively, and could be done either through a study of patient files or through direct observations. We opted for the latter, as it would also make it possible to observe whether the decision of continued treatment rested with the chiropractor or with the patient, or whether it was a joint decision.

Obviously, in the acute stage, decisions on the number and frequency of treatments would come mainly from the clinician. In the case of MC, however, one would expect more of a joint decision, or perhaps even that the patient requested continued care. In such a case, the use of MC can be looked upon as an active treatment, in which the patient takes responsibility and shows initiative to prevent new episodes of low back pain. However, if a long-term treatment program is imposed on patients, MC may become more of a passive ritual, removing the responsibility for keeping well from the patient to the treatment program. Such a strategy of passive coping may be detrimental for the prognosis [6]. For these reasons it is relevant to investigate if MC patients are more involved in the decision on the course of treatment than non-MC patients.

Therefore, it was the purpose of this study to investigate how chiropractic patients with low back pain were scheduled for treatment, with special emphasis on MC. The specific research questions were:

1. What is the proportion of patients on MC care in general chiropractic practice?

2. Are there specific patterns of intervals between treatments for patients and, if so, how do they differ between MC patients and non-MC patients?

3. Who decides on the next treatment, the patient, the chiropractor or both, and are there any differences between MC patients and non-MC patients?

## Methods

This was an observational study, in which data about the clinical encounter were collected on a pre-printed form by student observers. In addition, information about the chiropractors was collected in a self-report questionnaire.

The observation form and questionnaire were designed in April 2007. The questionnaire was written in Danish. To ensure user friendliness and enhance the likelihood of valid data, the forms were tested in a pilot study by the project leaders (CB and KFS) in two Danish chiropractic clinics. This resulted in some improvements of the wording of questions and layout.

Third year chiropractic students at the University of Southern Denmark collected the data during their obligatory one week clinical chiropractic practice period, which took place during their summer holiday of 2007 (June-August). Students would often return to their home during holidays, and they were allowed to observe clinics situated in Denmark, Norway or Sweden. There are no criteria set up by the university to approve clinics for observation. The clinicians can contact the university and volunteer to receive students or the students can contact a chiropractor of their choice (often in their hometown). The chiropractors receive a minor payment from the university for receiving the students. The Danish, Norwegian and Swedish languages are very similar, and no translation of the questionnaire would be necessary for participants outside of Denmark.

Data collection was voluntary and participation was encouraged at a meeting at which the project leaders informed their fellow students of the purpose of the study and the role of the students. The involved students attended a meeting where detailed verbal instruction was provided on how to proceed and how to fill out the observation forms. To standardize the discrimination between the three categories of decision-making, this was followed by role plays illustrating four imaginary chiropractic cases where an observation form was completed for each case. A translated observation form can be seen in Appendix 1.

Students would provide information on all patients seen on the days of observation. For each patient, information was collected about the previous visit and the next. There were five time intervals to choose from: 1) No new visit, 2) Next visit within one week, 3) Next visit between 2 and 4 weeks, 4) Next visit between 1-3 months, and 5) Next visit in 3 months or later. If the patient was a new patient to the clinic, this was noted. It was expected that patients would return at different time intervals depending on the duration since the last visit and that the time between visits would gradually increase.

The chiropractor was asked for each patient, whether (s)he could be considered to be a MC patient or not. When a new appointment was decided, the student made a judgement on whether this decision was made 1) mainly by the chiropractor, 2) mainly by the patient, or 3) whether it was more of a joint decision.

All data were collected anonymously and neither the chiropractor nor the patients could be identified. According to Danish law, there is no need for approval from an ethics committee for studies that do not include examination of individuals or human material.

The chiropractors were also asked to provide some demographic information (country of practice, gender, age, years of clinical experience, country in which they received their chiropractic education, size of town in which the clinic was located, and whether they were clinic owners or not). This information was used to compare the study sample with the target sample of chiropractors in the respective countries using information obtained from the national chiropractic associations. Particularly, educational background was considered to be important, as it was found in a previous study to predict attitudes to the use of MC (Signe F Hansen, Anne Line S Laursen, Tue S Jensen, Charlotte Leboeuf-Yde, Lise Hestbæk: The Nordic maintenance care program: what are the indications for maintenance care in patients with low back pain? A survey of the members of the Danish Chiropractors' Association, submitted).

To encourage participation of the chiropractors, an explanatory letter was sent out to the relevant clinics, with information about the study and an appeal for the chiropractors' co-operation.

When data collection was completed, the students returned the forms in a pre-stamped and addressed envelope to the main supervisor of the project. To motivate the students to send the data back, participants would be given a bottle of wine.

The data were analyzed manually from a spread-sheet. Demographic data were compared to information obtained from the chiropractic associations. Descriptive data were produced for each variable and information was compared for MC and non-MC patients. The differences between distributions were tested by means of Fisher's exact test.

All analyses were made separately for each country but combined if there were no obvious differences between the two. In order to study the appointment pattern, patients' past appointment was cross-tabulated against the next appointment, separately for MC patients and non-MC patients. To illustrate a possible difference in the decision-making between MC-patients and others, proportions were reported with 95% confidence interval.

Because our study sample turned out to be unrepresentative of its underlying study population (see result section) and the study sample of chiropractors was too small, no attempts were made to control for extraneous factors, such as school of graduation or age.

## Results

### Number of study participants

In all, 16 out of 30 students participated in our study. They collected data from 28 clinicians, 15 from Denmark, 13 from Norway (none from Sweden), but data from two Norwegian clinicians had to be omitted due to lack of information. In total, 868 patients were observed. Of these, 61 had to be excluded because of missing information. Fifty-six were new patients. According to the clinicians, 209 (26%) of the remaining patients were MC patients and 542 non-MC patients.

The range of clinicians per student was 1-4 and the range of patients per student was 15-119. The median number of patients observed by each student was 44 in Denmark and 50 in Norway. The range of patients observed for each clinician was 2-119 with a median of 30 in Denmark and 20 in Norway.

### Number of maintenance care patients

The range of MC patients per chiropractor in Denmark was 0%-50% and the mean and median values were 22%. The range was 0% - 100% among the Norwegian chiropractors, with a mean value of 26% and a median of 10%. The Norwegian group included two chiropractors with 0% MC patients and two with 93% and 100%, respectively. No such extreme values were seen for the Danish chiropractors.

### Description of the chiropractors and their representativeness

A comparison between the participants in the study and the underlying populations is shown in Table 1, with information provided for each country and for the two countries combined. Major differences are mentioned below.

**Table 1:** Demographic background of the chiropractors in the survey compared to the Danish Chiropractor's Association (DCA) and the Norwegian Chiropractor's Association (NCA).

	Danish participants n = 15	DCA n = 455	p	Norwegian participants n = 11	NCA n = 397	p	Total in survey	Total DCA and NCA	p
Sex									
- Female	67%	51%	0,297	9%	30%	0,187	42%	41%	1,000
- Male	33%	49%		91%	70%		58%	59%	

Age									
- 20-29	27%	6%	0,037	0%	16%	0,025	15%	11%	0,261
- 30-39	27%	31%		91%	48%		54%	39%	
- 40-49	20%	38%		0%	18%		12%	29%	
- 50-49	27%	18%		0%	15%		15%	16%	
- 60 or more	0%	7%		9%	3%		4%	5%	
- missing	-	-		-	<1%		-	<1%	

Country of graduation									
- DK	53%	33%	0,181	27%	11%	0,479	42%	23%	0,218
- UK	7%	22%		36%	37%		19%	29%	
- USA/Canada	40%	45%		36%	41%		39%	43%	
- Other	0%	<1%		0%	4%		0%	2%	
- missing	-	<1%		-	7%		-	3%	

Clinical experience (years)									
0-1	13%	7%	0,306	9%	-		12%	-	
2-5	27%	18%		7%	-		27%	-	
6-10	13%	10%		27%	-		19%	-	
11-19	7%	25%		27%	-		15%	-	
more than 20	40%	38%		9%	-		27%	-	
missing	-	2%		-	-		-	-	

Size of town/village									
0-20.000	7%	-		0%	-		4%	-	
20-100.000	33%	-		73%	-		50%	-	
> 100.000	60%	-		27%	-		46%	-	

Clinic owner/Employee									
- Clinic owner	67%	62%	0,793	82%	-		73%	-	
- Employee	3%	38%		18%	-		27%	-	

Reported in percentages and p-values for the difference between distributions, tested by means of Fisher's exact test.

There was an overrepresentation of female participants in Denmark compared to the gender distribution within the Danish profession but the opposite for the Norwegian participants.

The age of the participating Danish chiropractors differed somewhat from the general population of chiropractors. In Norway, the vast majority (91%) of the respondents were 30 to 39 years, which was almost twice as many as expected.

Half of the Danish participants were educated at the University of Southern Denmark, but only one-third of the Danish chiropractors belonged to this category. Similar differences were noted for the Norwegian chiropractors.

In relation to years of clinical experience, there were almost twice as many as expected in the Danish study group with 0-1 year of clinical experience as compared to the whole profession and the group with a clinical experience of 11-19 years was underrepresented (7% vs. 25%). This comparison could not be done for the Norwegian chiropractors.

The majority of the Danish participants practised in towns of more than 100.000 inhabitants whereas the majority of the Norwegian participants were found in towns of 20.000-100.000 inhabitants. No comparison could be made with the study populations.

The percentages of clinic owners and employees corresponded well with the underlying Danish population. This information was missing in relation to Norway (Table 1).

In summary, the two study samples deviated considerably from the underlying study population on several variables and, notably, on the most important variable, namely country of graduation. The study sample of chiropractors can therefore not be considered to be representative of its target group.

### Are there specific patterns of intervals between treatments for patients and, if so, do they differ between MC patients and non-MC patients?

As can be seen in Table 2, for non-MC patients the largest group consisted of patients who had their last visit within one week, and of these, 63% would be booked for a new visit again within one week. In fact, regardless of when the last visit took place, the most common choice was to re-schedule again within 1 week. The second most common choice was to give no new appointment, presumably because some patients were "cured" and very few <1% would be given a new appointment in 3 months time or more.

**Table 2:** The time period for the next treatment by the time period for the last treatment for non-maintenance care patients in a survey of chiropractors in Denmark and Norway.

DK+N	No new visit (n = 108)	Next visit within 1 week (n = 297)	Next visit between 2-4 weeks (n = 84)	Next visit between 1-3 months (n = 51)	Next visit in over 3 months or later (n = 2)	Total
Last visit within 1 week (n = 342)	17%	**63%**	15%	4%	0%	100%

Last visit within 2-4 weeks (n = 107)	21%	**31%**	23%	23%	1%	100%

Last visit within 1-3 moths (n = 44)	27%	**41%**	11%	18%	2%	100%

Last visit within 3 months or later (n = 49)	29%	**61%**	4%	6%	0%	100%

The bold print show percentage of time-intervals which were most commonly used.

Table 3 shows how, for the MC patients, the last visit most commonly occurred within the past 2-4 weeks, or within the past 1-3 months. There were two equally large groups who were last seen within 1 week or within 3 months or later.

**Table 3:** The time period for the next treatment by the time period for the last treatment of maintenance care patients in a survey of chiropractors in Denmark and Norway.

DK+N	No new visit (n = 20)	Next visit within 1 week (n = 42)	Next visit between 2-4 weeks (n = 41)	Next visit between 1-3 months (n = 80)	Next visit in over 3 months or later (n = 26)	Total
Last visit within 1 week (n = 37)	3%	**51%**	27%	16%	3%	100%

Last visit within 2-4 weeks (n = 72)	10%	15%	**32%**	**40%**	3%	100%

Last visit within 1-3 moths (n = 62)	13%	13%	6%	**58%**	10%	100%

Last visit within 3 months or later (n = 38)	11%	11%	11%	24%	**45%**	100%

The bold print show percentage of time-intervals which were most commonly used.

Contrary to the non-MC patients, the re-scheduling of MC-patients depended on when the last visit occurred. Those last seen within 1 week would again be booked within 1 week (51%), those last seen within 2-4 weeks would be seen again within the same time interval (32%) or within 1-3 months (40%). Those last seen within 1-3 months would again be scheduled in 1-3 months (58%) and those who came at least 3 months ago would do so again (45%). The most commonly selected interval for next visit was between 1 and 3 months.

### Who decides on the next treatment, the patient, the chiropractor or both, and are there any differences between MC patients and non-MC patients?

For both MC patients and non-MC patients the chiropractors in our study would be the primary initiators in relation to the subsequent treatment. The estimates were higher for the Danish chiropractors than for the Norwegian chiropractors. For the Norwegian participants, it was almost as common that both chiropractor and patient were involved with this decision. This was far less common among the Danish chiropractors. Among the Danish chiropractors, a higher degree of patient influence was noted among the MC-patients than among the non-MC patients, with 34% and 20%, respectively, involved in the decision about the next visit. A similar pattern was not detected in Norway (Table 4).

**Table 4:** Table describing who takes the initiative for the next appointment, the chiropractor, the patient or both in a survey of maintenance and non-maintenance care patients treated by Danish and Norwegian chiropractors.

DENMARK Initiative taken by	Maintenance care patient (n = 103)	Non-maintenance care patients (n = 318)
Chiropractor	61% (51-71%)	74% (69-79%)

Patient	7% (3-14%)	4% (2-7%)

Both	27% (19-37%)	16% (12-21%)

Missing	5% (2-11%)	6% (4-9%)

	100%	100%

**NORWAY** **Initiative taken by**	**Maintenance care patient (n = 106)**	**Non-maintenance care patients (n = 224)**

Chiropractor	47% (37-57%)	47% (40-54%)

Patient	10% (5-18%)	9% (6-13%)

Both	43% (2-11%)	40% (34-47%)

Missing	0%	4% (2-7%)

	100%	100%

Percentage (95% confidence interval)

## Discussion

We found that 22% of the patients in Denmark and 26% of the patients in Norway were on maintenance care, illustrating the need to take this aspect of care seriously. In a survey among all practising chiropractors in Denmark with a response rate of 72%, the proportion of MC patients was 22% (Signe F Hansen, Anne Line S Laursen, Tue S Jensen, Charlotte Leboeuf-Yde, Lise Hestbæk: The Nordic maintenance care program: what are the indications for maintenance care in patients with low back pain? A survey of the members of the Danish Chiropractors' Association, submitted). This indicates that although our sample of chiropractors is not representative, at least the Danish part of the sample is representative in this aspect. We are not aware of similar investigations in Norway.

This appears to be the first study to have looked at the time frame between visits for patients with low back pain attending chiropractic clinics, and also the first study to look at the point of initiative for the subsequent visit. We found that there were two distinct patterns in how new visits are scheduled. For non-MC patients a new appointment would often be booked within one week whereas there were more possibilities for MC patients. These possibilities seemed to depend on when the previous visit occurred.

From our study, it is impossible to know whether this was a dynamic pattern, i.e. that these patients were booked with different intervals depending on their clinical development, or a static one, i.e. that patients were booked repeatedly with identical treatment intervals.

The most commonly used interval for MC patients was 1-3 months. Longer intervals were much less common, in fact almost as uncommon as no new visit.

We were surprised to note that the patients of the Danish chiropractors in this study had so little say in the course of their treatment, although the MC patients did have a slightly higher degree of influence than the acute patients, as we expected. The Norwegian participants were also found to favour the paternalistic approach, but it was almost equally as common in this group that both the chiropractor and the patient took part in the decision of the next appointment.

However, these results may not necessarily be extrapolated to other groups of chiropractors. The reason for this is that the chiropractors of our study appeared not to be representative of the chiropractors in Denmark and Norway.

This lack of representativeness is perhaps not surprising. The chiropractors who participated in the study accepted that students observed them during their work. Graduates from the Danish university course would probably be more inclined to accept students from that same place, which would explain the educational skewness in our study sample. Educational background has previously been shown to have an effect on Danish chiropractors' attitude to MC (Signe F Hansen, Anne Line S Laursen, Tue S Jensen, Charlotte Leboeuf-Yde, Lise Hestbæk: The Nordic maintenance care program: what are the indications for maintenance care in patients with low back pain? A survey of the members of the Danish Chiropractors' Association, submitted). This is likely to have biased our results in unknown direction.

Not all of the eligible students accepted to help with the study. It is, of course, possible that also this could have resulted in a bias, if participating students were more interested in MC and/or recorded the data inaccurately. However, this is not very likely, because the data recording left little room for subjectivity.

A likely source of error, however, is the time of year, when data were collected. This took place during the summer holidays, a period with less activity in many clinics, and probably with an over representation of patients in acute pain. The proportion of no new visits may also have been inflated, if they emanated from tourists, who consulted a chiropractor for emergency assistance. Therefore, the proportion of MC patients in the involved practices may be larger during the rest of the year. The day of the week or the time of the day, when data were collected, may also have affected the ratio of non-MC patients and MC patients. For example, in Denmark, many chiropractors rarely book MC patients on a Monday, when emergency cases are expected, and some chiropractors even set aside certain times on certain days for that type of patients.

Despite the weaknesses of this study, it also has some strengths. Data were based on observations rather than subjective estimates. The forms and questionnaires were standardized to make it possible to compare and analyse data from the different clinics and students had been thoroughly instructed in how to use the forms and how to return them, thus minimizing the risk of data collection errors. In a future study, patient files will be examined retrospectively to establish the time pattern of visits.

## Conclusion

In this particular group of chiropractors, MC was used for about one quarter of the patients, ranging from 0% to 100% between clinics. The intervals with which the chiropractors saw their MC patients were distinctly different from that of their non-MC patients. An interval of 1-3 months for the next appointments was most frequently used for MC patients but this depended on the duration since the last treatment. In most cases, the observing student considered that the chiropractor and not the patient took the initiative in arranging the next appointment, regardless of whether it was a MC patient or a non-MC patient. However, it is not known, if these results can be extrapolated to other groups of chiropractors and other types of patients.

## Competing interests

The authors declare that they have no competing interests.

## Authors' contributions

CLY and LH were responsible for conception and design, CB and KFS carried out the data collection, all authors contributed to data analysis and interpretation, CLY and LH drafted the manuscript. All authors read and approved the final manuscript.

## Information about authors

This study was a part requirement for the MSc degree in health science (biomechanics), at the University of Southern Denmark, Odense, Denmark for CB and KFS.
